# Overnight fasting before lapatinib administration to breast cancer patients leads to reduced toxicity compared with nighttime dosing: a retrospective cohort study from a randomized clinical trial

**DOI:** 10.1002/cam4.3528

**Published:** 2020-10-23

**Authors:** Moe Tsuda, Hiroshi Ishiguro, Naoko Toriguchi, Norikazu Masuda, Hiroko Bando, Masahiro Ohgami, Masato Homma, Satoshi Morita, Naohito Yamamoto, Katsumasa Kuroi, Yasuhiro Yanagita, Toshimi Takano, Satoru Shimizu, Masakazu Toi

**Affiliations:** ^1^ Department of Breast Surgery Graduate School of Medicine Kyoto University Kyoto Japan; ^2^ Breast Oncology Service Saitama Medical University International Medical Center Hidaka Japan; ^3^ Oncology Products Medicine Development Unit Japan Eli Lilly Japan K.K Kobe Japan; ^4^ Department of Surgery, Breast Oncology NHO Osaka National Hospital Osaka Japan; ^5^ Breast and Endocrine Surgery Faculty of Medicine University of Tsukuba Tsukuba Japan; ^6^ Department of Pharmacy Ibaraki Prefectural Central Hospital Kasama Japan; ^7^ Department of Pharmaceutical Sciences Division of Clinical Medicine Faculty of Medicine University of Tsukuba Tsukuba Japan; ^8^ Department of Biomedical Statistics and Bioinformatics. Graduate School of Medicine Kyoto University Kyoto Japan; ^9^ Division of Breast Surgery Chiba Cancer Center Chiba Japan; ^10^ Department of Breast Surgery Tokyo Metropolitan Health and Hospitals Corporation Ebara Hospital Tokyo Japan; ^11^ Department of Breast Oncology Gunma Prefectural Cancer Center Gunma Japan; ^12^ Breast Medical Oncology Breast Oncology Center The Cancer Institute Hospital of JFCR Tokyo Japan; ^13^ Breast and Endocrine Surgery Kanagawa Cancer Center Yokohama Japan

**Keywords:** breast cancer, drug discovery and delivery, medical oncology, quality of life, Tyrosine kinase inhibitors

## Abstract

**Background:**

The bioavailability of lapatinib is affected by food, even following the 1 hour fast recommended by the package insert. We hypothesized that overnight fasting would minimize food‐drug interactions. Here, we investigated if lapatinib administration timing is associated with its tolerability, efficacy, and pharmacokinetics.

**Methods:**

This is a retrospective cohort study utilizing the medical records of patients enrolled in the JBCRG‐16/Neo‐LaTH randomized phase 2 trial for breast cancer patients treated with lapatinib. Lapatinib administration timing was divided into three groups: before breakfast (BB), between meals (BM), and at bedtime (AB). Side effects (SE), treatment discontinuation rate (TDR), relative dose intensity (RDI), pathological complete response (pCR) rate, and lapatinib serum trough concentration were compared between groups.

**Results:**

About 140 patients were included in this study: BB 15, BM 51, and AB 74. A reduced risk of diarrhea {adjusted hazard ratio (HR), 0.51, 95% confidence interval (CI), 0.27‐0.89, *p* = 0.018}, and rash {adjusted HR, 0.37; 95% CI, 0.17‐0.70, *p* = 0.002} was seen in BB versus AB. Fewer patients with low RDI (< 0.85/<0.6) were in the BB group (BB 13% / 0%, BM 22% / 3.9%, AB 24% / 14%, *p* = 0.70 / 0.11). pCR was not diminished (*p* = 0.75). BB group had the lowest serum lapatinib concentration and variability (mean ±SD were 0.35 ± 0.15, 0.65 ± 0.32, 0.96 ± 0.43 µg/ml).

**Conclusions:**

Compared to bedtime administration, lapatinib administration after overnight fasting reduces its toxicity without diminishing its therapeutic efficacy.

## Graphical abstract

The bioavailability of lapatinib is affected by food, even when following package insert information. In a retrospective cohort study that utilized the medical records of 140 patients enrolled in the JBCRG‐16/Neo‐LaTH randomized phase 2 trial for breast cancer who were treated with lapatinib, associations between the timing of lapatinib administration (before breakfast, between meals or at bedtime) and clinical outcomes such as toxicities likely due to lapatinib (diarrhea, skin rash and hepatotoxicity) and drug efficacy were evaluated. Lapatinib administration after overnight fasting reduced the incidence of drug‐related diarrhea and skin rash without diminishing its therapeutic efficacy by avoiding food‐drug interactions.

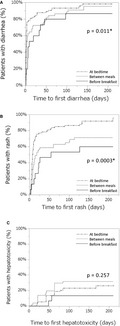

## 1 BACKGROUND

Lapatinib (Tykerb®, GlaxoSmithKline, Brentford United Kingdom) is an oral antihuman epidermal growth factor receptor (HER2) and anti‐epidermal growth factor receptor (EGFR) tyrosine kinase inhibitor (TKI) that is used in combination with other antineoplastic drugs. In the setting of metastatic breast cancer, lapatinib 1250‐1500 mg is effective and well‐tolerated in combination with capecitabine or letrozole.^1^, ^2^ In the NeoALTTO trial, adding 750 mg of lapatinib to the standard trastuzumab and paclitaxel neoadjuvant regimen nearly doubled the pathological complete response (pCR) rate (51.3% vs 29.5%), although 18‐21% of patients could not complete the lapatinib‐containing treatment as planned due to intolerable toxicity.^3^ Since lapatinib is an oral agent, toxicity is not simply dose dependent. Different recommended dosages based on drug combination seem to be partially due to drug‐drug interactions. Importantly, the bioavailability of lapatinib is very vulnerable to the presence of food. A 4.25‐fold the area under the blood drug concentration‐time curve (AUC), the actual body exposure to drug after administration of a dose of the drug was observed when lapatinib was administrated with a high‐fat diet while an 2.67‐fold AUC was observed with low‐fat intake.^4^ The package insert recommends that lapatinib be taken on an empty stomach “at least 1 hour before‐ or after‐ a meal.” This recommendation was not modified despite a later company‐sponsored trial showed that even if lapatinib was administered 1 hour after a meal as per the package insert recommendation, its resulting serum concentration was two times higher than if it was taken after an overnight fast.^5^

This food‐drug interaction is one of the largest among the oral TKIs. The next largest effect is a 1.6‐fold increase reported for erlotinib after 2 hours of recommended fasting, and many other TKIs with smaller food effects are recommended to be taken after 2‐3 hours of fasting.^4^ We questioned if the 1 hour fasting recommendation for lapatinib is enough to avoid its interaction with food. Here, we performed a retrospective cohort study using data from the Neo‐LaTH / JBCRG‐16 randomized phase II clinical trial of primary HER2 positive breast cancer patients treated with neoadjuvant lapatinib to investigate if the timing of lapatinib administration is associated with its toxicity, pharmacokinetic stability, dose modification, and therapeutic efficacy.

## 2 METHODS

### 2.1 Study design of Neo‐LaTH / JBCRG‐16

The Neo‐LaTH / JBCRG‐16 trial, which was previously reported by N. Masuda et al,^6^ was a randomized phase II registration clinical trial that evaluated the efficacy and safety of lapatinib and trastuzumab therapy followed by lapatinib, trastuzumab, and weekly paclitaxel in Japanese patients with primary HER2 positive breast cancer. A total of 213 patients were enrolled between March 2012 and September 2013 and randomized into groups A and B (Estrogen receptor: ER ‐), and C‐E (ER +). Patients received neoadjuvant lapatinib (1000 mg/d) and trastuzumab for 6 weeks (A, C, and D) or 18 weeks (B and E). All groups then received 12 weeks of lapatinib (750 mg/d), trastuzumab and weekly paclitaxel (80 mg/m^2^). Group D and E received additional endocrine therapy.

### 2.2 Eligibility criteria, endpoint, and statistical analysis

The timing of lapatinib administration was obtained from the medical records of all 213 patients enrolled in Neo‐LaTH / JBCRG‐16, and three groups were formed: 1) before breakfast administration, 2) between meals administration, and 3) administration at bedtime. Patients without a clear description of dose timing in their medical record were excluded. Patients who changed their dose timing during the course of their treatment were also excluded from this analysis.

Targeted adverse events of interest were diarrhea, rash, and hepatotoxicity as classified by the National Cancer Institute Common Terminology Criteria for Adverse Events (CTCAE) version 4.0. A rash was defined as an “acne like rash,” which is the classically and most commonly seen skin reaction to EGFR inhibitors. Hepatotoxicity was defined as the elevation of any one of alanine aminotransferase (ALT) or aspartate aminotransferase (AST), alkaline phosphatase (ALP), or bilirubin. Information about regular or preventive use of antidiarrheals, laxatives, and moisturizers is also obtained.

Descriptive statistics included counts and proportions for categorical variables and mean, standard deviation (SD), and range for continuous variables. Categorical variables were compared with the use of the chi‐squared test or Fisher's exact test. Continuous variables were compared with a one‐way analysis of variance (ANOVA). Unadjusted estimates of the risk of adverse events at any grade were compared with the Kaplan‐Meier method and the Wilcoxon test. The adjusted estimate of risk factors for adverse events was computed with the Cox proportional hazard regression model. In order to keep the risk of overfitting low, three variables (i.e., administration timing, age, and duration of lapatinib treatment) were selected based on previous reports or clinical relevance; younger patients has been reported to develop more rash and less diarrhea, and additional cycle has been reported to cause diarrhea less frequently.^7^, ^8^ pCR was defined as ypT0 / Tis. Relative dose intensity (RDI) of lapatinib was defined as the ratio of delivered dose intensity to the dose intensity of the planned dose. Delivered dose intensity was defined as total delivered dose divided by actual time to complete chemotherapy or standard time to complete chemotherapy, whichever was longer. Lapatinib serum trough concentrations were measured with high‐performance liquid chromatography (HPLC) as previously described^9^ in patients who agreed to separate informed consent, and blood samples were collected at 24 ± 2 hours after the last dose on day 22. Levene's test followed by Welch's *t* test was used to compare serum concentrations of lapatinib; *p* < 0.025 was considered statistically significant. All statistical analyses were performed using JMP Software (version 12; SAS Institute, Cary, NC). This study was approved by the Institutional Review Board and followed the guidelines for observational studies.

## 3 RESULTS

### 3.1 Patients characteristics

**Figure 1:**
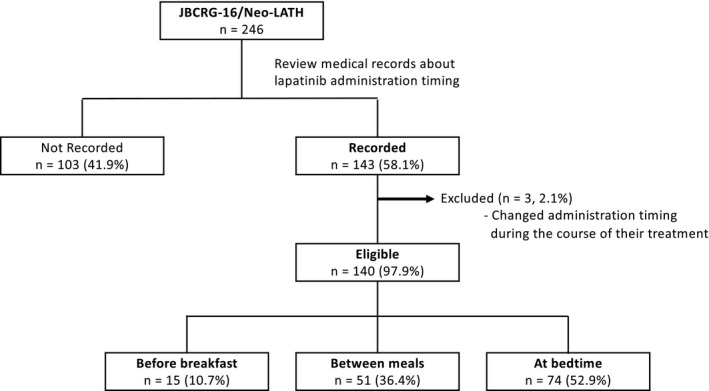
Flow diagram of this study

Out of 213 patients in the JBCRG‐16 / Neo‐LaTH trial, lapatinib administration timing was obtained from medical records for 143 (67.5%). Three (2.1%) patients were excluded because the timing of their lapatinib administration was changed during the course of their treatment (Figure 1). The characteristics of the remaining 140 patients were similar with those of the overall patients in the JBCRG‐16 / Neo‐LaTH trial (Table 1). Of the 140 patients, 15 (10.7%) received lapatinib before breakfast, 51 (36.4%) between meals, and 74 (52.9%) at bedtime. Overall patient characteristics were similar between the three groups (Table 2). Only small number of patients used preventive medications regularly.

**Table 1:** Comparison of patients’ characteristics by inclusion of this study

		Patients in this study (n = 140)	Overall patients in the JBCRG−16/Neo‐LaTH trial (n = 213)	*p* value
Age (y)	Mean ± SD (range)	53.5 ± 10.3 (26‐69)	53.0 ± 10.0 (26‐70)	0.68
Menopause status	Pre Post	56 (40%) 84 (60%)	91 (43%) 122 (57%)	0.61
BMI	Mean ± SD (range)	22.8 ± 0.33 (14.7‐37.4)	22.5 ± 0.27 (14.1‐37.4)	0.43
Regimen	A	28 (20%)	44 (21%)	0.91
	B	28 (20%)	48 (23%)	
	C	26 (19%)	41 (19%)	
	D	32 (23%)	40 (19%)	
	E	26 (19%)	40 (19%)	
Diarrhea (any grade)		132 (94%)	199 (93%)	0.83
Rash (any grade)		112 (80%)	166 (78%)	0.64
Hepatotoxicity (any grade)		36 (26%)	54 (25%)	0.94
pCR		69 (49%)	101 (47%)	0.73

**Table 2:** Patients’ characteristics by the timing of lapatinib administration

		Before breakfast (n = 15)	Between meals (n = 51)	At bedtime (n = 74)	*p* value
Age (y)	Mean ± SD (range)	55.7 ± 11.0 (31‐69)	52.9 ± 9.6 (28‐68)	52.3 ± 10.8 (26‐68)	0.51
Menopause status	Pre	3 (20%)	28 (55%)	44 (59%)	0.22
	Post	12 (80%)	23 (45%)	30 (41%)	
BMI	Mean ± SD (range)	21.4 ± 3.4	22.7 ± 3.6	23.2 ± 4.1	0.25
		(14.7‐26.2)	(15.9‐33.2)	(17.3‐37.4)	
Regimen	A	5 (33%)	9 (18%)	14 (19%)	0.49
	B	2 (13%)	9 (18%)	17 (23%)	
	C	2 (13%)	8 (16%)	16 (22%)	
	D	3 (20%)	17 (33%)	12 (16%)	
	E	3 (20%)	8 (16%)	15 (20%)	
Lapatinib 1000 mg	6 weeks	10 (67%)	34 (67%)	42 (57%)	0.52
	18 weeks	5 (33%)	17 (33%)	32 (43%)	
Estrogen receptor	Positive	8 (53%)	33 (65%)	43 (58%)	0.65
	Negative	7 (47%)	18 (35%)	31 (42%)	
Stage	II	7 (47%)	21 (41%)	42 (57%)	0.22
	III	8 (53%)	30 (59%)	32 (43%)	
Regular/preventive medication	Laxatives	0 (0%)	6 (12%)	8 (11%)	0.55
	Antidiarrheals	0(0%)	1 (2%)	1 (1%)	1
	Moisturizers	3 (20%)	2 (4%)	6 (8%)	0.11

Neoadjuvant treatment regimen with lapatinib (1000 mg/d) and trastuzumab for 6 weeks (A, C, and D) or 18 weeks (B and E) before receiving 12 weeks of lapatinib (750 mg/d), trastuzumab plus weekly paclitaxel (80 mg/m^2^) treatment. Group D and E received additional endocrine therapy.

### 3.2 Association with adverse events

**Figure 2:**
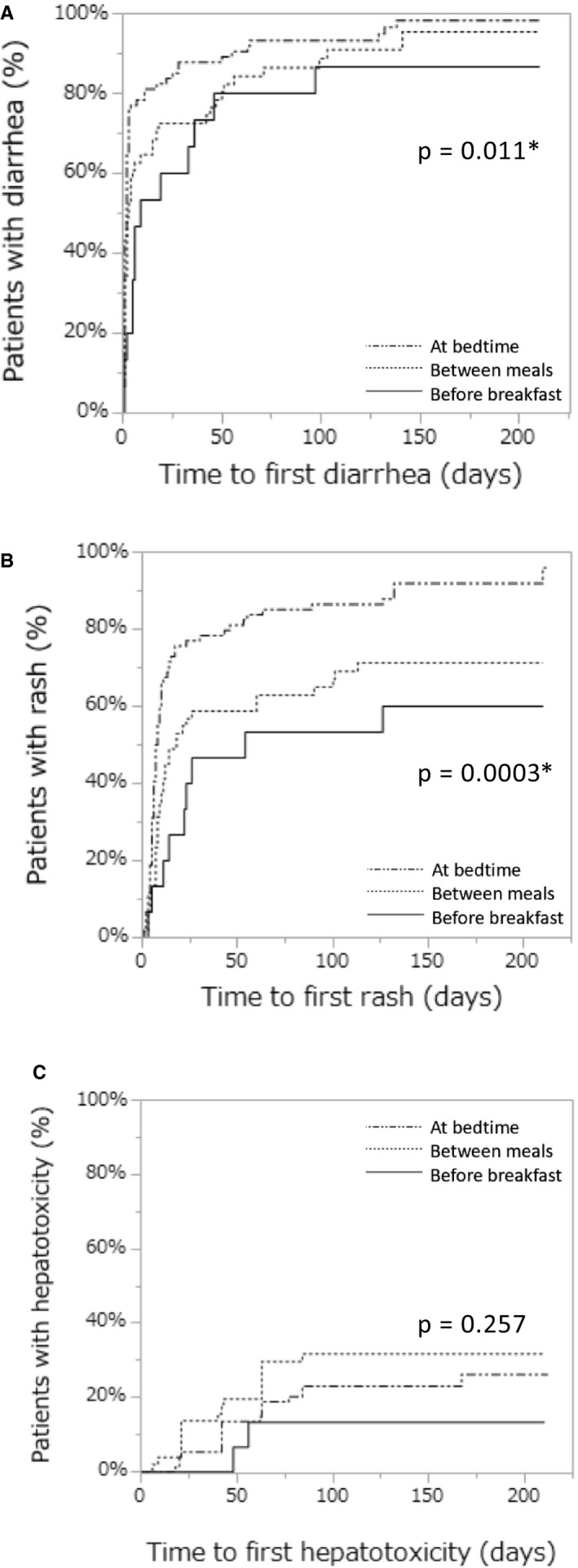
A Kaplan‐Meier plot of the proportion of patients without diarrhea (A), a rash (B), and hepatotoxicity (C)

Figure 2 shows Kaplan‐Meier cumulative incidence estimates of the time to the first occurrence of three targeted adverse events, with *p* values calculated with the Wilcoxon test. Diarrhea at any grade occurred in 13 / 15 (86.7%) patients in the before breakfast group, 47 / 51 (92.2%) in the between meals group, and 72 / 74 (97.3%) in the at bedtime group. A rash was observed in 9 / 15 (60.0%), 36 / 51 (70.6%), and 67 / 74 (90.5%) patients, respectively. Hepatotoxicity was observed in 2 / 15 (13.3%), 16 / 51 (31.4%), and 18 / 74 (24.3%) patients, respectively (Table 3). Univariate and multivariate Cox regression analysis revealed that the risks of both diarrhea and rash were lower in the before breakfast group compared with the at bedtime group (diarrhea: unadjusted hazard ratio [HR], 0.54; 95% confidence interval [CI], 0.28‐0.94, *p* = 0.028, and adjusted HR, 0.51, 95% CI, 0.27‐0.89, *p* = 0.018, rash: unadjusted HR 0.36; 95% CI, 0.17‐0.69, *p* = 0.001, and adjusted HR, 0.37; 95% CI, 0.17‐0.70, *p* = 0.002). Similarly, the risk of a rash was significantly lower in the between meals group compared with the at bedtime group (unadjusted HR, 0.52; 95% CI, 0.34‐0.78, *p* = 0.001, and adjusted HR, 0.53; 95% CI, 0.35‐0.80, *p* = 0.002). An age <55 was associated with a significantly higher risk of a rash compared to patients ≥55 years old (unadjusted HR, 1.49; 95% CI, 1.03‐2.17, *p* = 0.036, and adjusted HR, 1.46; 95% CI, 1.01‐2.13, *p* = 0.046). No independent risk factor for hepatotoxicity was identified, although a lower tendency toward developing hepatotoxicity was observed in the before breakfast group compared to the between meals and the at bedtime groups (Table 4).

**Table 3:** Frequency of adverse events according to administration timing

	Before breakfast (n = 15)	Between meals (n = 51)	At bedtime (n = 74)	*p* value
Diarrhea				
Any grade	13 (86%)	47 (92%)	72 (97%)	0.12
Grade 3/4	2 (13%)	3 (6%)	3 (4%)	0.30
Rash				
Any grade	9 (60%)	36 (71%)	67 (91%)	0.003*
Grade 3/4	1 (7%)	1 (2%)	1 (1%)	0.34
Hepatotoxicity				
Any grade	2 (13%)	16 (31%)	18 (24%)	0.16
Grade 3/4	0 (0%)	1 (2%)	5 (7%)	0.38

**Table 4:** Hazard ratio for the three targeted adverse events calculated by Cox regression model.

	Variable	Univariate			Multivariate		
		HR	95% CI	*p* value	HR	95% CI	*p* value
Diarrhea	Timing						
	BB vs BM	0.72	0.37‐1.30	0.285	0.7	0.36‐1.26	0.244
	BB vs AB	0.54	0.28‐0.94	0.028	0.51	0.27‐0.89	0.018
	BM vs AB	0.74	0.51‐1.07	0.114	0.73	0.50‐1.05	0.089
	Age (y)						
	<55 vs ≧55	0.84	0.59‐1.18	0.312	0.83	0.59‐1.18	0.297
	Duration^#^						
	18 vs 6 weeks	0.84	0.58‐1.20	0.336	0.8	0.55‐1.14	0.22
Rash	Timing						
	BB vs BM	0.69	0.31‐1.38	0.31	0.69	0.31‐1.37	0.3
	BB vs AB	0.36	0.17‐0.69	0.001	0.37	0.17‐0.70	0.002
	BM vs AB	0.52	0.34‐0.78	0.001	0.53	0.35‐0.80	0.002
	Age (y)						
	<55 vs ≧55	1.49	1.03‐2.17	0.036	1.46	1.01‐2.13	0.046
	Duration^#^						
	18 vs 6 weeks	1.19	0.81‐1.74	0.369	1.1	0.75‐1.61	0.624
Hepatotoxicity	Timing						
	BB vs BM	0.37	0.06‐1.32	0.138	0.37	0.06‐1.30	0.134
	BB vs AB	0.53	0.08‐1.83	0.349	0.53	0.08‐1.85	0.359
	BM vs AB	1.41	0.71‐2.77	0.323	1.44	0.72‐2.84	0.3
	Age (y)						
	<55 vs ≧55	0.6	0.30‐1.15	0.123	0.59	0.30‐1.14	0.119
	Duration^#^						
	18 vs 6 weeks	1.07	0.53‐2.09	0.836	1.12	0.55‐2.20	0.745

Abbreviations: AB, At bedtime; BB, Before breakfast; BM, Between meals; CI, confidence interval; HR, hazard ratio.

^#^ Lapatinib duration before paclitaxel.

### 3.3 Association with treatment discontinuation, dose reduction, and efficacy

The rate of treatment discontinuation (1 / 15 (6.7%) before breakfast, 7 / 51 (13.7%) between meals, 16 / 74 (20.3%) at bedtime, *p* = 0. 23) was numerically lower in the before breakfast group and an RDI of less than 0.85, which is generally considered to be a clinically meaningful threshold for reduction on previously published studies,^10^ was seen in 2 / 15 (13.3%), 11 / 51 (21.6%), 18 / 74 (24.3%), respectively (*p* = 0.70). Extremely low RDI (defined as RDI <0.6) was seen in 0 / 15 (0%), 2 / 51 (3.9%), and 10 / 74 (13.5%), respectively (*p* = 0.11). The pCR rate was similar between the three groups (8 / 15 (53.3%) before breakfast, 23 / 51 (45.1%) between meals, and 38 / 74 (51.4%) at bedtime, *p* = 0.75).

### 3.4 Association with serum trough concentrations

**Figure 3:**
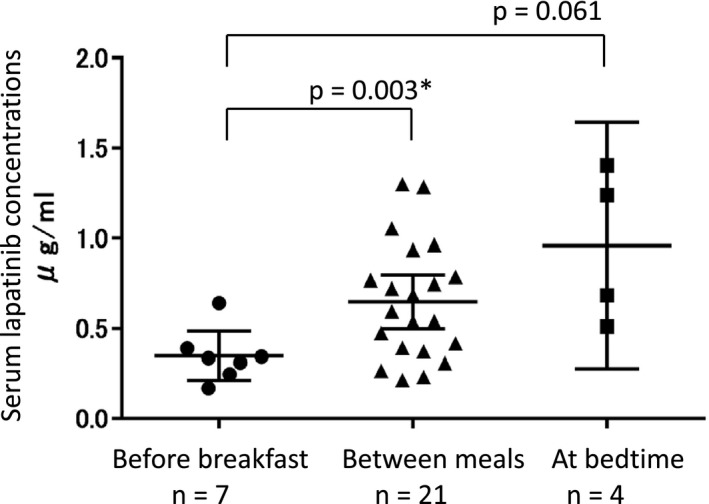
Serum trough lapatinib concentrations

Lapatinib serum trough concentrations were measured in 36 out of 140 patients. Four (11.1%) were excluded because of an inappropriate blood collection time after the last drug administration. We could obtain trough blood sample of at bedtime group from only four patients out of 74 patients participated in this study. The before breakfast group had the lowest serum lapatinib concentrations and its variability, and the difference was statistically significant when compared to between meals group (mean ± SD were 0.35 ± 0.15, 0.65 ± 0.32, 0.96 ± 0.43 µg/ml, coefficient variation [CV] were 42.7%, 50.1%, 44.8%, respectively. *p* = 0.024 by Levene's test, and *p* = 0.003 [vs. between meals], *p* = 0.061 [vs at bedtime] by Welch's *t* test) (Figure 3).

## 4 DISCUSSION

Many patients fail to complete lapatinib treatment as planned because of toxicities.^3^ It is well known that lapatinib bioavailability is influenced by prandial conditions. In this study, patients who took lapatinib before breakfast (after overnight fasting) had a lower incidence of diarrhea and a rash compared with those who took lapatinib at bedtime. Patients who received lapatinib before breakfast had approximately half the risk of developing diarrhea and about a third of the risk of developing a rash compared with patients taking lapatinib at bedtime, which was the most preferred administration time in this study. Our results suggest that the “1 hour fast” recommended in the package insert does not avoid food‐lapatinib interactions.

This study has several limitations because this is an unplanned analysis from a prospectively conducted trial using subgroups of patients classified by the timing of their lapatinib administration. First, only 67.5% of patients in the JBCRG‐16 trial were able to be enrolled in this analysis due to data availability about lapatinib administration since this recoding was not mandatory. We did not conduct any additional interview survey consciously. Instead, we collected information from existing medical records with the use of a systematic form of chart abstraction in order to avoid recall and interviewer biases, which preferably provided almost equal chance of inclusion to all patients. Furthermore, we used the database of a randomized registration clinical trial, where toxicities would be more accurately and consistently documented than that achieved in clinical practice. As a result, this study is at a relatively small risk of information and selection biases despite that limited number of patients were enrolled. Second, as is typical of this type of study, only association and not causation can be inferred from our result because it is subject to confounding. For example, the bedtime group could have more concomitant medications such as sleeping pills, which could affect lapatinib absorption and/or metabolism via cytochrome P450 3A4. This is difficult to control since there are good reasons for taking these medications at night regardless of interaction. It is also possible that circadian rhythm (biological clock) affected biochemical reactions; several experimental and clinical studies^11^, ^12^, ^13^ have reported positive associations between the circadian clock and drug response in cancer patients. Such confounding, if any, could be also reflected in real world clinical practice.

Little is presently known about the risk factors for lapatinib‐induced toxicities. Dose and pharmacokinetic exposure are, however, certainly responsible. The magnitude of the food‐drug interaction must differ from day to day and person to person because we eat different dishes at different times, which will contribute to intra‐ and inter‐patient variability after lapatinib exposure. Taking lapatinib after an overnight fast is the best way to control for intra‐ and inter‐patient variability by minimizing food‐drug interaction. The mechanisms by which food increases lapatinib bioavailability are, however, complicated and poorly understood. Delayed gastric emptying and increased pH are considered to play little role, because tablet dissolution time is sufficiently rapid (6‐minute half‐life in vitro) and higher pH results in lower solubility of lapatinib especially in pH 4‐5 where gastric pH is supposed to be after ingestion of meal,^4^ Alternatively, many other factors contribute to the great magnitude on the increase of bioavailability; for example, increased blood flow to the intestinal mucosa, prolonged small intestinal transit time, stimulated bile flow (particularly with a high‐fat food), and activated food‐induced enzymes, such as CYP 3A4.^4^ Regretfully, we did not investigate the individual eating habits or the exact fasting times before lapatinib administration or whether patients in the before breakfast group ate breakfast. Therefore, it remains unclear which food types are responsible for the food‐drug interaction, or how much time would be needed to avoid it. Based on previous work, which reported a 1.8‐fold increase in AUC after consuming low‐fat food versus a 2.6‐fold increase after consuming high‐fat food when lapatinib was administrated after a 1 hour fast,^5^ the magnitude of the Japanese‐food effect observed in this single‐country study predicted to be smaller than that of Western‐food because the median fat intake of a Japanese female (50.0 grams per day) is lower than that of an American female (71.5 g/d). However, trough serum lapatinib concentration of between meal group was 1.9 times higher compared to that of before breakfast group. Our earlier preliminary report including 15 patients from this trial and six from others^14^ also showed that trough lapatinib concentration was four times higher with 1 hour after meal administration compared to 1 hour before meal administration.

Determining the optimal dosing for an individual patient is difficult but very important, especially for anticancer agents. In an initial phase 3 trial before the FDA approval of lapatinib, lapatinib was taken 1 hour before or after breakfast. No reasonable evidence supports the 1 hour fasting rule currently recommended by the package insert at any time of day. The magnitude of the lapatinib's food‐drug interaction is much bigger than any of its currently known drug‐drug interactions. A 20% increase in serum concentration has been reported when lapatinib is used in combination with paclitaxel, and a 26% decrease is reported when it is used in combination with proton pump inhibitors.^15^ Although recent clinical trials for early stage breast cancer patients used 750 mg/d of lapatinib in combination with paclitaxel, this dosing is not based on efficacy but tolerability. Earlier studies reported that 900 to 1200 mg of lapatinib was needed to produce a clinical response.^16^ Lapatinib at 1500 mg/day combined with paclitaxel showed clinical benefit with moderate tolerability in earlier phase 3 trials for metastatic breast cancer and gastric cancer.^17^, ^18^ Based on our findings there is some room for dose escalation, especially if dosed before breakfast group, with the goal of improving clinical efficacy.

Although a rash induced by other EGFR TKIs (e.g., erlotinib or gefitinib) has been predictive of favorable clinical efficacy in lung, pancreas, and head and neck cancers,^19^, ^20^, ^21^, ^22^ it remains unclear if a lapatinib‐induced rash is associated with treatment efficacy. The GeparQuinto neoadjuvant trial reported no association between rash and pCR rate,^23^ the Neo ALTTO trial reported a limited association only in elderly patients with a “early rash” that develops within 6 weeks of the start of therapy,^7^ and the ALTTO trial reported a positive association between an “early rash” and survival.^24^ In breast cancer, however, EGFR is not the principal therapeutic target and is not highly co‐expressed with HER2.^7^ Therapeutic drug monitoring can reduce drug exposure variability and has been shown to be useful in predicting the efficacy of imatinib for chronic myeloid leukemia,^25^ but with a modest budget impact.^26^

In conclusion, lapatinib administration before breakfast can minimize the effects of food on lapatinib concentration and reduce the incidence of diarrhea and a rash compared with at bedtime dosing. This approach is easy to apply with no added cost or inconvenience to patients, and has the potential to permit dose escalation.

## CONFLICT OF INTEREST

HI, NM, KK, TT, and MaT are the member of directors for Japan Breast Cancer Research Group as nonfinancial competing interests. MoT reports grants from JSPS KAKENHI Grant Number JP17 J09462, outside the submitted work. HI reports grants from Japan Breast Cancer Research Group and MEXT KAKENHI Grant Number JP24590656, during the conduct of the study. NT has nothing to disclose. NM reports grants, personal fees and other from Chugai, personal fees and other from AstraZeneca, personal fees and other from Pfizer, personal fees and other from Elli‐Lilly, grants, personal fees and other from Eisai, personal fees and other from Takeda, personal fees and other from Kyowa‐Kirin, other from MSD, personal fees and other from Novartis, personal fees and other from Daiichi Sankyo, outside the submitted work. HB reports personal fees from AstraZeneca, personal fees from Eisai, personal fees from Kyowa Kirin, personal fees from Taiho, personal fees from Chugai, personal fees from Nihon Kayaku, personal fees from Pfizer, personal fees from Novartis, outside the submitted work. MO reports personal fees from Chugai, personal fees from Daiichi Sankyo, personal fees from Ono, personal fees from Mochida, outside the submitted work. MH has nothing to disclose. SM reports personal fees from AstraZeneca, personal fees from Bristol‐Myers Squibb, personal fees from Chugai, personal fees from Eisai, personal fees from Eli Lilly, personal fees from MSD, personal fees from Pfizer, personal fees from Taiho, outside the submitted work. NY reports grants from Japan Breast Cancer Research Group, during the conduct of the study; grants from MSD, grants from Pfizer, grants from Eli Lilly, grants from Nihon Kayaku, outside the submitted work. KK reports other from Kyowa Kirin, other from Eisai, outside the submitted work. YY has nothing to disclose. TT reports grants and personal fees from Daiichi‐Sankyo, grants and personal fees from Chugai, grants and personal fees from Kyowa Kirin, grants and personal fees from Eisai, grants from Ono, grants from BMS, grants from MSD, grants from Merck Serono, grants from Taiho, grants from Novartis, personal fees from Pfizer, personal fees from Eli Lilly, personal fees from Celltrion Healthcare, outside the submitted work. SS has nothing to disclose. MaT reports grants and personal fees from Chugai, grants and personal fees from Takeda, grants and personal fees from Pfizer, grants, personal fees and other from Kyowa‐Kirin, grants and personal fees from Taiho, grants from JBCRG association, grants and personal fees from Eisai, grants, personal fees, and other from Daiichi‐Sankyo, grants and personal fees from AstraZeneca, personal fees from Eli Lilly, personal fees from MSD, personal fees from Genomic Health, personal fees from Novartis, personal fees and other from Konica Minolta, grants from Astellas, other from BMS, grants and personal fees from Shimadzu, personal fees from Yakult, grants and personal fees from Nihon Kayaku, grants from AFI technologies, other from Athenex Oncology, outside the submitted work, and Board of directors for Organization for Oncology and Translational Research, Kyoto Breast cancer Research Network.

## AUTHOR CONTRIBUTIONS

Conceptualization: MoT, HI, NT, NM, MO, MH, SM, and MaT. Data curation: all. Formal analysis: MoT, HI, NT, NM, MO, MH, SM, and MaT. Funding acquisition: HI, NM, HB, NY, KK, YY, TT, SS, MH, and MaT. Investigation: MoT, HI, NT, NM, HB, NY, KK, YY, TT, SS, MO, MH, and MaT. Methodology: MoT, HI, NT, MO, MH, SM, and MaT. Project administration: HI, NM, MH, SM, and MaT. Resources: MoT, HI, NT, NM, MH, SM, and MaT. Supervision: HI, MH, SM, and MaT. Validation: HI, NM, MH, SM, and MaT. Visualization: MoT, HI, NM, MO, MH, SM, and MaT. Writing‐original draft: MoT and HI. Writing‐review and editing: All authors. All authors approved final version of the manuscript to be published. All authors agreed to be accountable for all aspects of the work to ensure that questions related to the accuracy or integrity of any part of the work are appropriately investigated and resolved.

## Data Availability

The data that support the findings of this study are available from the corresponding author, upon reasonable request.
